# Macromolecule suppressed GABA levels show no relationship with age in a pediatric sample

**DOI:** 10.1038/s41598-020-80530-8

**Published:** 2021-01-12

**Authors:** Tiffany Bell, Mehak Stokoe, Ashley D. Harris

**Affiliations:** 1 Department of Radiology, University of Calgary, Calgary, AB Canada; 2 Hotchkiss Brain Institute, University of Calgary, Calgary, AB Canada; 3 Alberta Children’s Hospital Research Institute, University of Calgary, 28 Oki Drive, Office B4-510, Calgary, AB T3B 6A9 Canada

**Keywords:** Neurochemistry, Neuroscience, Development of the nervous system

## Abstract

The inhibitory neurotransmitter γ-Aminobutyric acid (GABA) plays a crucial role in cortical development. Therefore, characterizing changes in GABA levels during development has important implications for the study of healthy development and developmental disorders. Brain GABA levels can be measured non-invasively using GABA-edited magnetic resonance spectroscopy (MRS). However, the most commonly used editing technique to measure GABA results in contamination of the GABA signal with macromolecules (MM). Therefore, GABA measured using this technique is often referred to as GABA+ . While few in number, previous studies have shown GABA+ levels increase with age during development. However, these studies are unable to specify whether it is specifically GABA that is increasing or, instead, if levels of MM increase. In this study, we use a GABA-editing technique specifically designed to suppress the MM signal (MM-supp GABA). We find no relationship between MM-supp GABA and age in healthy children aged 7–14 years. These findings suggest that the relationship between GABA+ and age is driven by changes in MM levels, not by changes in GABA levels. Moreover, these findings highlight the importance of accounting for MM levels in MRS quantification.

## Introduction

γ-Aminobutyric acid (GABA) is the primary inhibitory neurotransmitter in the human brain and plays a crucial role in mediating neuronal activity, cortical inhibition and plasticity^1^. As a result, GABA has an essential role in multiple cortical functions including a direct role in cortical development through synaptic pruning^2^. Therefore, changes in GABA levels during development are important to define, not only to understand typical development, but also to provide context to understand alterations in neurodevelopmental disorders (i.e., autism spectrum disorder, ASD, attention deficit hyperactivity disorder, ADHD) as well as clinical conditions, particularly those with symptoms or prodromal symptoms in childhood and adolescence, such as migraine and psychosis.

Proton magnetic resonance spectroscopy (MRS) is the only method to non-invasively measure brain metabolites in vivo. Due to its relatively low abundance in the brain and the overlap of more abundant metabolite signals on the spectrum, GABA levels are typically measured using the MEscher-GArwood editing sequence, MEGA-PRESS^3^. Briefly, this involves applying a frequency selective editing pulse at 1.9 ppm in half of the spectra (‘ON’ sub-spectra) which specifically refocuses the coupling of the 3.0 ppm GABA peak. Interleaved with this, measurements are taken with no editing pulse applied (“OFF” sub-spectra). Subtraction of the ON spectra from the OFF spectra removes the overlapping signals that were not modulated by the editing pulse in order to resolve a GABA peak at 3 ppm.

However, the editing pulse applied to the 1.9 ppm GABA resonance is not perfectly selective and co-edits a macromolecule (MM) peak at 1.7 ppm which is also coupled to a 3.0 ppm resonance. As a result, the 3 ppm GABA peak is contaminated with an MM signal by ~ 50%^4,5^. Therefore this measurement is often referred to as GABA+ to represent GABA + MM. One approach to limit the MM contamination is to apply an editing pulse during the edit-OFF portion of the experiment at 1.5 ppm. This is symmetric to the 1.9 ppm GABA editing pulse around the 1.7 ppm MM peak^6,7^. In this approach, both the ON and the OFF sub-spectra equally affect (or edit) the coupled MM signal and it is removed in the difference spectrum. The 1.5 ppm pulse is far enough away from the 1.9 ppm GABA resonance that it is not affected making this editing pulse specific to the MM signal^7^. Additionally, the editing pulses used in this acquisition are longer and therefore more selective (20 ms vs 14 ms in a GABA+ Acquisition). To ensure clarity, in this manuscript this measurement of GABA will be referred to as MM-supp GABA. For more details on the technical implementation and comparisons of GABA+ and MM-supp acquisitions, readers are referred to ^4,6,8^.

Prior work has shown GABA+ increases during development^9,10^, followed by a decline late in life^11,12^ though it is possible this decline may be due to natural tissue atrophy^13,14^. However, others have not replicated this same relationship^15^, which may be a result of a small age-range or the location of the voxel. It has been shown that GABA+ levels vary across the brain^16,17^, with GABA+ levels tending to increase moving from an anterior to a posterior location, therefore age-related changes may also vary across the brain.

Alternatively, due to the contamination of MM within the GABA+ measurement, it is possible that MM influence or drive the age-related increases in GABA+ . Indeed, Aufhaus et al. (2013) demonstrated a relationship between GABA+ and age in subjects aged 20–50 years that was not seen when using MM-supp GABA measurements^18^, suggesting MM changes drive the relationship between GABA+ and age in young-middle aged adults. It is therefore likely that MM levels have an effect on age-related changes in children as well.

The aim of the present study is to examine the relationship between age and MM-supp GABA levels in children aged 7–14 years in three locations in the brain; the thalamus, sensorimotor cortex and occipital cortex. These areas represent a range of GABA levels and cortical functions in the brain and are some of the most commonly reported regions in the MRS literature. This is the first study to specifically examine the relationship of MM-supp GABA and age in a pediatric cohort.

## Methods

### Ethics statement

The study protocol was approved by the Conjoint Health Research Ethics Board (CHREB), University of Calgary. All experiments were performed in accordance with CHREB guidelines and regulations. All of the study participants provided informed assent and their parents provided informed consent at time of enrollment.

### Participants

Children aged 7 to 14 years were recruited using the Healthy Infants and Children Clinical Research Program (HICCUP). Participants were included if they had no history of developmental, neurological or psychiatric disorder and met the standard MRI safety criteria (e.g. no metal implants or devices).

### MRI acquisition

**Figure 1:**
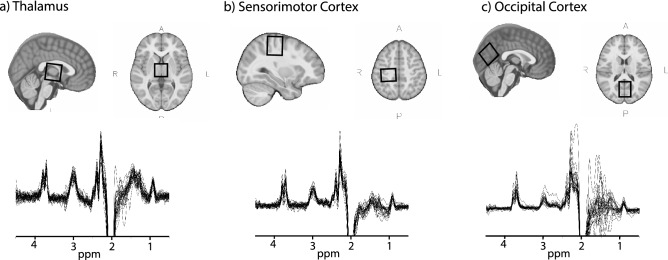
Example voxel placement and spectra from all participants from (**a**) Thalamus, (**b**) Sensorimotor cortex and (**c**) Occipital cortex.

Imaging was performed on a 3 T scanner (750w, General Electric Healthcare) and included a T1-weighted imaging acquisition (BRAVO: TE/TR = 2.7/7.4 ms, slice = 1 mm^3^ isotropic voxels) that was used for voxel placement and subsequent voxel segmentation for tissue correction. MM-suppressed GABA-edited spectroscopy data (TR/TE = 1.8 s/80 ms, 20 ms editing pulses at 1.9 ppm and 1.5 ppm, 256 averages, voxel size 3 × 3 × 3 cm^3^) and 8 unsuppressed-water averages were collected from the thalamus (midline centered), right sensorimotor cortex (centered on the hand-knob of the motor cortex, and rotated such that the coronal and sagittal planes aligned with the cortical surface^19^) and the occipital cortex (as close to aligning with the parietoocciptal sulcus as possible, without including cerebellum, midline centered, Fig. 1).

### MRI analysis

Data were processed using Gannet 3.1^20^, including retrospective frequency and phase correction using spectral registration^21^, voxel segmentation and tissue specific water relaxation^22^, with removal of the typically applied “MM correction factor (MM = 0.45)” as applied in Gannet by default. Data were visually inspected, and rejected if unresolvable subtraction artifacts were present, typically resulting from motion.

We report quantified MM-supp GABA in molal units, accounting for tissue specific relaxation and mole fractions of water as per standard MRS quantification approaches^23^. As secondary and follow-up analyses, we report MM-supp GABA including the “α-correction”^22^, which accounts for the higher concentration of GABA in grey matter (GM) compared to white matter (WM) (α = 0.5, which assumes the concentration of GABA in GM is twice that of WM). As a confirmatory analysis, we quantify MM-supp GABA relative to creatine (MM-supp GABA/Cr) to allow comparison to previous studies and ensure results were not due to water signal differences.

### Statistical analysis

Statistical analyses were performed using SPSS (IBM. 2017. IBM SPSS Statistics for Macintosh, Version 25.0. Armonk, NY: IBM). Using the Shapiro–Wilk test, the majority of variables were assessed to be normally distributed, with only MM-supp GABA and MM-supp GABA (α-corrected) not normally distrusted. For consistency, and as parametric tests have more power than non-parametric tests, parametric tests were used for all comparisons. Non-parametric tests were also run for comparisons concerning MM-supp GABA and MM-supp GABA (α-corrected), however this did not change the result and therefore are not reported here.

MM-supp GABA levels (using the three quantification methods) and the voxel GM ratio (calculated as fGM/[fGM + fWM], where fGM and fWM are the voxel fractions of grey matter and white matter, respectively), were compared between the three locations using repeated measures ANOVAs. Where Mauchly’s test of sphericity showed the assumption of sphericity to be violated, the Greenhouse–Geisser correction is applied (noted as F_gg_). Effect sizes are reported as partial eta-squared (η_p_^2^).

For correlation analyses, age was calculated in months, however for visualization age is presented in years. For each of the three voxels, correlations of MM-supp GABA vs age (in months), MM-supp GABA (α-corrected) vs age and MM-supp GABA/Cr vs age were estimated using Pearson’s correlation coefficient. Additionally, this analysis was repeated for MM-supp GABA and MM-supp GABA/Cr using partial correlations controlling for the GM ratio in each voxel. MM-supp GABA (α-corrected) was not included in this secondary analysis as the GM ratio is already included in the α-correction.

## Results

Data was acquired from 29 children and adolescents (mean age 10.17 years, SD 1.79, 14 males, 15 females). Data from the sensorimotor cortex voxel from two children was excluded due to poor quality, otherwise all spectra were included (Thalamus: N = 29; Sensorimotor Cortex: N = 27; Occipital Cortex: N = 29).

**Figure 2:**
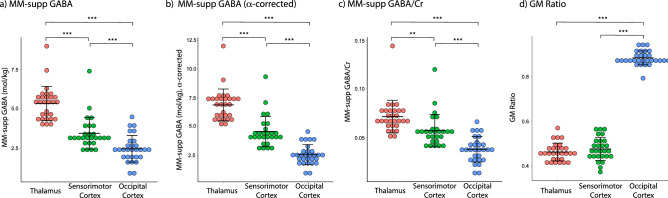
Regional differences in MM-Supp GABA levels and GM ratio. (**a**) MM-supp GABA. (**b**) MM-supp GABA (α-corrected). (**c**) MM-supp GABA/Cr. (**d**) GM ratio. Lines represent mean ± standard deviation. **p < 0.01, ***p < 0.001.

As seen in adults, there was a significant effect of voxel location on MM-supp GABA levels (MM-supp GABA: F_gg_(1.5,39.8) = 67.8, p < 0.001, η_p_^2^ = 0.723; MM-supp GABA α-corrected: F(2,52) = 94.3, p < 0.001 η_p_^2^ = 0.784; MM-supp GABA/Cr: F(2,52) = 29.6, p = 0.04, η_p_^2^ = 0.532) and the GM ratio (F(2,52) = 1121.1, p < 0.001 η_p_^2^ = 0.977). MM-supp GABA levels (mol/kg and mol/kg α-corrected) were significantly higher in the thalamus than the sensorimotor cortex (p < 0.001) and the occipital cortex (p < 0.001), and MM-supp GABA levels (mol/kg and mol/kg α-corrected) in the sensorimotor cortex were significantly higher than in the occipital cortex (p < 0.001). MM-supp GABA/Cr levels were significantly higher in the thalamus (p < 0.001) and sensorimotor cortex (p < 0.001) than the occipital cortex, and significantly higher in the thalamus than the sensorimotor cortex (p = 0.01). The GM ratio was significantly lower in the thalamus (p < 0.001) and sensorimotor cortex (p < 0.001) than the occipital cortex, there was no significant difference in the GM ratio between the thalamus and sensorimotor cortex (Fig. 2).

**Figure 3:**
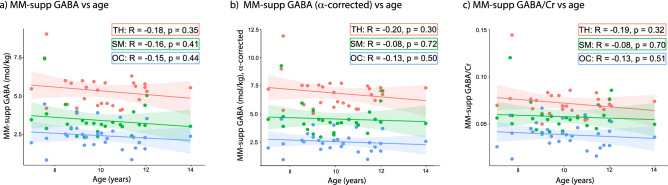
Correlations between the three versions of MM-supp GABA levels and age. (**a**) Correlation between MM-supp GABA and age. (**b**) Correlation between α-corrected MM-supp GABA and age. (**c**) Correlation between MM-supp GABA/Cr and age. TH: Thalamus (red); SM: Sensorimotor cortex (green); OC: Occipital cortex (blue).

There were no significant correlations between age and MM-supp GABA levels in any of the three regions in both the primary analysis or the secondary analyses using the α-correction or GABA/Cr (Fig. 3; Table 1).

**Table 1:** Correlations between MM-supp GABA measures and age (months).

		Thalamus	Sensorimotor Cortex	Occipital Cortex
MM-supp GABA vs age		r(28) = − 0.18, p = 0.35	r(26) = − 0.16, p = 0.41	r(28) = − 0.15, p = 0.44
MM-supp GABA (α-corrected) vs age		r(28) = − 0.20, p = 0.30	r(26) = − 0.08, p = 0.72	r(28) = − 0.13, p = 0.50
MM-supp GABA/Cr vs age		r(28) = − 0.19, p = 0.32	r(26) = 0.08, p = 0.70	r(28) = − 0.13, p = 0.51
Controlling for GM ratio	MM-supp GABA vs age	r(26) = − 0.18, p = 0.35	r(24) = − 0.28, p = 0.17	r(26) = − 0.24, p = 0.22
	MM-supp GABA/Cr vs age	r(26) = − 0.18, p = 0.37	r(24) = − 0.24, p = 0.24	r(26) = − 0.25, p = 0.21

**Figure 4:**
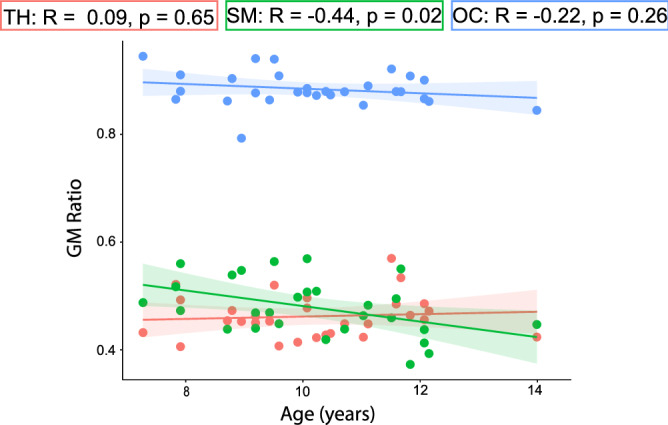
Correlation between GM ratio (fGM/[fGM + fWM]) and age. TH: Thalamus (red); SM: Sensorimotor cortex (green); OC: Occipital cortex (blue).

There was a significant correlation between the GM ratio and age in the sensorimotor cortex only (r(27) =  − 0.44, p = 0.02; Fig. 4). However, controlling for the GM ratio did not change the relationship between MM-Supp GABA and age in any region (Table 1).

## Discussion

Several studies have shown that GABA+ increases with age during development (e.g. ^9,10^), however this measurement is contaminated with roughly 50% MM^5^. It is often assumed that MM levels are stable, but there has been no attempt to directly validate this in development. Here, we use a sequence specifically designed to suppress the MM signal that contaminates the measured GABA+ signal when using MEGA-PRESS. We show no relationship between MM-supp GABA and age in children aged 7–14 years.

In adults, it has been proposed that age-related changes in GABA+ are mediated by tissue changes, specifically decreases in GM^13^. In the current study, tissue-specific relaxation factors and tissue-voxel content were accounted for in the primary analysis, following conventions in the field^23,24^. As it has been shown that the concentration of GABA is higher in GM compared to WM^22^, we performed secondary analyses; (1) using α-corrected GABA levels, and (2) controlling for the GM-tissue fraction using partial correlations^22^. The α-correction assumes twice as much GABA is present in GM compared to WM, however, it should be noted that this was determined using adult data and has yet to be validated in children. Consistent with our primary result, neither of these analyses indicate a relationship between MM-Supp GABA and age. Finally, to facilitate comparison with previous studies as well as confirm that the reference water signal has no influence on the results, GABA/Cr was analyzed and showed no relationship with age.

Our results suggest that the previously seen relationship between GABA+ and age during development is in fact driven by age-related changes in MM, though this would need to be confirmed using MM specific sequences such as metabolite nulling. Indeed, macromolecule levels have been shown to increase with age in adult rats^25^. In humans, older adults have been shown to have higher levels of MM than younger adults^26,27^, further studies are needed to determine if this is applicable throughout the lifespan. Interestingly, we found levels of MM-supp were lowest in the occipital cortex of children aged 7–14, whereas previous studies have shown levels of GABA+ in the occipital cortex tend to be higher than other areas^9,17^. This difference may again be the result of MM contamination. Indeed, MM levels themselves^28,29^ and the correlation between GABA + and MM-supp GABA^4^ have shown to vary across the brain, indicating MM levels show inter- and intra-subject variability.

MM signals seen in MRS spectra represent amino acids with flexible polypeptide chains, cytosolic proteins and mobile lipids. Changes in levels of MM have been suggested to represent multiple aspects of cell turnover, such as cell proliferation and apoptosis, inflammation, necrosis, and perturbations of membrane turnover^30^. Developmental changes in MM may reflect changes in metabolic turnover, though the specific mechanism cannot be elucidated from this study. However, changes in MM levels have important implications for using MRS, and specifically here using GABA+-edited MRS, to study development in typical and atypical populations. Indeed, though it is often assumed that MM levels remain stable, this has been shown to be inaccurate in several diseases. For example, MM levels have been shown to be elevated in multiple sclerosis, possibly due to cleavage of myelin proteins into smaller, less rigid polypeptides, or oligodendrocyte pathology^31^. Additionally, increases in MM have been seen after stroke, suggested to represent increased visibility of cytosolic proteins after cell death^32^. MM levels have also been shown to be decreased in premature neonates, which was interpreted as a reduction in protein and mobile lipid synthesis^33^. Mikkelsen et al. (2018) showed strong correlations between MM-supp GABA and behavioral measures that were no longer significant when using GABA+ measures^34^. This implies that variability in MM levels can mask changes in GABA levels. Not only does this highlight that MM should be appropriately controlled for during metabolite quantification, but this also highlights that MM levels themselves should be studied as potential biomarkers of disease mechanisms.

GABA is synthesized by the enzyme glutamic acid decarboxylase (GAD), of which there are two isoforms GAD65 and GAD67. GAD65 is localized in the axon terminals and synthesizes the on-demand pool of GABA (vesicular GABA). GAD65 is phasically active and plays a role in inhibitory synaptic transmission. GAD67 is tonically active and is hypothesized to have a role in metabolism. GAD67 is located in the cell body and synthesizes the basal pool of GABA (cytoplasmic GABA)^35,36^. It is thought that MRS mainly measures the GABA levels in this basal pool^1,37^. In the visual cortex, the expression of GAD65 has been shown to vary across the lifespan (increases during development and declines during old age), whereas the expression of GAD67 remains stable, indicating the basal pool of GABA is maintained across the lifespan^35^. Extracellular GABA concentration modulates rates of GABA reuptake, therefore any changes in GABA levels caused by changes in the expression of GAD65 will be matched by either alterations in GABA synthesis or vesicular release by the activation of presynaptic GABA autoreceptors or changes in GABA recycling at the synapses, resulting in no visible change in the overall basal pool of GABA, and subsequently no change in MRS measured GABA^38^. This provides support for our results of no change in MM-supp GABA levels during development, whilst also highlighting the limitations of MRS-measured GABA.

The relationship between levels of GABA measured by MRS and GABAergic function in the brain is incompletely understood. MRS-measured GABA is often described as measuring “inhibitory tone”, the extent to which a region can exert inhibition^1^. The relationship between local inhibition and function will depend on local cortical cytoarchitecture and neuronal circuitry. For example, the application of a GABAergic agonist enhances tonic inhibition in layers 2/3 and 5 of the neocortex^39^ but increases excitation in layer 4 circuits^40^. Therefore higher tonic GABA levels may not necessarily reflect higher inhibition. This is likely due to the fact that tonic GABA levels affect both the excitability of principal neurons and also interneuronal activity. Interneurons communicate with one another as well as with numerous principal neurons, further complicating interpretation^41^. However, multiple studies have shown relationships between GABA+ and behavioural measures of inhibition^15,42–48^. Therefore, MRS-measured GABA likely reflects some form of inhibition, however further studies are needed to elucidate the relationship between GABA levels and inhibition.

Despite the contamination of MM, using a GABA+ MEGA-PRESS sequence does have some advantages over a MM-supp MEGA-PRESS sequence. The more selective pulses needed for MM-supp MEGA-PRESS require a longer echo time (TE = 80 ms for MM-supp GABA, TE = 68 ms for GABA+) and are more sensitive to frequency drift, although drift will change the level of MM contamination of GABA+ measures^49^. Additionally, the MM-supp GABA signal is roughly 50% smaller than the GABA+ signal and as a result has a lower SNR and higher fit error^8,34,50^. Therefore, the choice of MM-supp GABA vs GABA+ will depend on study specific factors such as the region of interest and the maximum acceptable scan time. Never-the-less, studies using GABA+ should be cautious of their interpretations as this study evidences that MM may have a more significant contribution than often assumed.

A limitation of this study is the lack of direct MM measurement. Therefore, we cannot directly confirm that the lack of correlation between MM-supp GABA and age in this cohort is due to the absence of MM signal. As mentioned previously, the more selective MM-supp pulses are more sensitive to frequency drift and MM-supp GABA measures have lower SNR and higher fit error^8,34^. The mean data drift in this study was 10.24 Hz (SD = 7.68 Hz) and the mean fit error was 7.22 (SD = 2.54). However, results herein could be confirmed using a metabolite nulled measure. Additionally, as with the majority of studies of this nature, this study has a cross sectional design. A longitudinal design would more accurately track changes in GABA and MM levels during development. MRS measures in general also suffer from a lack of spatial specificity, due to the large voxel size (needed for sufficient SNR to quantify GABA). The balance between SNR and regional specificity is a particular issue for pediatric data, due to their generally smaller brain size. It is possible that a more spatially specific voxel would detect a different relationship between age and MM-supp GABA levels in children.

In conclusion, we found no relationship between age and MM-supp GABA in children age 7–14 years across three voxels in the brain. Given prior suggestions in the literature that GABA+ increases with age, we suggest increases in MM levels may drive previously shown relationships between age and GABA+ levels.

## Data Availability

The dataset generated and analysed during the current study are available from the corresponding author on reasonable request.

## References

[1] Rae CD (2014). A guide to the metabolic pathways and function of metabolites observed in human brain 1H magnetic resonance spectra. Neurochem. Res..

[2] Wu X (2012). GABA signaling promotes synapse elimination and axon pruning in developing cortical inhibitory interneurons. J. Neurosci..

[3] Mescher M, Merkle H, Kirsch J, Garwood M, Gruetter R (1998). Simultaneous in vivo spectral editing and water suppression. NMR Biomed..

[4] Harris AD, Puts NAJ, Barker PB, Edden RAE (2015). Spectral-editing measurements of GABA in the human brain with and without macromolecule suppression. Magn. Reson. Med..

[5] Harris AD, Saleh MG, Edden RAE (2017). Edited ^1^H magnetic resonance spectroscopy in vivo: Methods and metabolites. Magn. Reson. Med..

[6] Henry PG, Dautry C, Hantraye P, Bloch G (2001). Brain gaba editing without macromolecule contamination. Magn. Reson. Med..

[7] Edden RAE, Puts NAJ, Barker PB (2012). Macromolecule-suppressed GABA-edited magnetic resonance spectroscopy at 3T. Magn. Reson. Med..

[8] Mikkelsen M, Singh KD, Sumner P, Evans CJ (2016). Comparison of the repeatability of GABA-edited magnetic resonance spectroscopy with and without macromolecule suppression. Magn. Reson. Med..

[9] Gaetz W (2014). GABA estimation in the brains of children on the autism spectrum: measurement precision and regional cortical variation. Neuroimage.

[10] Saleh MG (2020). Effect of age on GABA+ and glutathione in a pediatric sample. Am. J. Neuroradiol..

[11] Gao F (2013). Edited magnetic resonance spectroscopy detects an age-related decline in brain GABA levels. Neuroimage.

[12] Porges EC (2017). Frontal gamma-aminobutyric acid concentrations are associated with cognitive performance in older adults. Biol. Psychiatry Cogn. Neurosci. Neuroimaging.

[13] Maes C (2018). Age-related differences in GABA levels are driven by bulk tissue changes. Hum. Brain Mapp..

[14] Porges EC (2017). Impact of tissue correction strategy on GABA-edited MRS findings. Neuroimage.

[15] Puts NAJ (2017). Reduced GABA and altered somatosensory function in children with autism spectrum disorder. Autism Res..

[16] Durst CR (2015). Noninvasive evaluation of the regional variations of GABA using magnetic resonance spectroscopy at 3 tesla. Magn. Reson. Imaging.

[17] Grewal M (2016). GABA quantitation using MEGA-PRESS: regional and hemispheric differences. J. Magn. Reson. Imaging.

[18] Aufhaus E (2013). Absence of changes in GABA concentrations with age and gender in the human anterior cingulate cortex: a MEGA-PRESS study with symmetric editing pulse frequencies for macromolecule suppression. Magn. Reson. Med..

[19] Yousry TA (1997). Localization of the motor hand area to a knob on the precentral gyrus. A new landmark. Brain.

[20] Edden RAE, Puts NAJ, Harris AD, Barker PB, Evans CJ (2014). Gannet: A batch-processing tool for the quantitative analysis of gamma-aminobutyric acid-edited MR spectroscopy spectra. J. Magn. Reson. Imaging.

[21] Near J (2014). Frequency and phase drift correction of magnetic resonance spectroscopy data by spectral registration in the time domain. Magn. Reson. Med..

[22] Harris AD, Puts NAJ, Edden RAE (2015). Tissue correction for GABA-edited MRS: considerations of voxel composition, tissue segmentation, and tissue relaxations. J. Magn. Reson. Imaging.

[23] Near J (2020). Preprocessing, analysis and quantification in single-voxel magnetic resonance spectroscopy: experts’ consensus recommendations. NMR Biomed..

[24] Gasparovic C (2006). Use of tissue water as a concentration reference for proton spectroscopic imaging. Magn. Reson. Med..

[25] Fowler C, Madularu D, Dehghani M, Devenyi G, Near J (2020). Longitudinal quantification of metabolites and macromolecules reveals age- and sex-related changes in the healthy fischer 344 rat brain. bioRxiv.

[26] Marjańska M (2018). Altered macromolecular pattern and content in the aging human brain. NMR Biomed..

[27] Hofmann L, Slotboom J, Boesch C, Kreis R (2001). Characterization of the macromolecule baseline in localized 1H-MR spectra of human brain. Magn. Reson. Med..

[28] Mader I (2002). Proton magnetic resonance spectroscopy with metabolite nulling reveals regional differences of macromolecules in normal human brain. J. Magn. Reson. Imaging.

[29] Považan M (2018). Simultaneous mapping of metabolites and individual macromolecular components via ultra-short acquisition delay 1 H MRSI in the brain at 7T. Magn. Reson. Med..

[30] Hakumäki JM, Kauppinen RA (2000). 1H NMR visible lipids in the life and death of cells. Trends Biochem. Sci..

[31] Mader I (2001). Proton MR spectroscopy with metabolite-nulling reveals elevated macromolecules in acute multiple sclerosis. Brain.

[32] Saunders DE, Howe FA, Van Den Boogaart A, Griffiths JR, Brown MM (1997). Discrimination of metabolite from lipid and macromolecule resonances in cerebral infarction in humans using short echo proton spectroscopy. J. Magn. Reson. Imaging.

[33] Koob M (2016). Creatine, glutamine plus glutamate, and macromolecules are decreased in the central white matter of premature neonates around term. PLoS ONE.

[34] Mikkelsen M, Harris AD, Edden RAE, Puts NAJ (2018). Macromolecule-suppressed GABA measurements correlate more strongly with behavior than macromolecule-contaminated GABA+ measurements. Brain Res..

[35] Pinto JGA, Hornby KR, Jones DG, Murphy KM (2010). Developmental changes in GABAergic mechanisms in human visual cortex across the lifespan. Front. Cell. Neurosci..

[36] Stagg CJ, Bachtiar V, Johansen-Berg H (2011). What are we measuring with GABA magnetic resonance spectroscopy?. Commun. Integr. Biol..

[37] Schmidt-Wilcke T (2018). GABA—from inhibition to cognition: emerging concepts. Neuroscientist.

[38] Myers JF, Nutt DJ, Lingford-Hughes AR (2016). γ-aminobutyric acid as a metabolite: Interpreting magnetic resonance spectroscopy experiments. J. Psychopharmacol..

[39] Drasbek KR, Jensen K (2006). THIP, a hypnotic and antinociceptive drug, enhances an extrasynaptic GABAA receptor-mediated conductance in mouse neocortex. Cereb. Cortex.

[40] Krook-Magnuson EI, Huntsman MM (2005). Excitability of cortical neurons depends upon a powerful tonic conductance in inhibitory networks. Thalamus Relat. Syst..

[41] Stagg CJ (2014). Magnetic resonance spectroscopy as a tool to study the role of GABA in motor-cortical plasticity. Neuroimage.

[42] Puts NAJ, Edden RAE, Evans CJ, McGlone F, McGonigle DJ (2011). Regionally specific human GABA concentration correlates with tactile discrimination thresholds. J. Neurosci..

[43] Hermans L (2018). GABA levels and measures of intracortical and interhemispheric excitability in healthy young and older adults: a MRS-TMS study. Neurobiol. Aging.

[44] Quetscher C (2015). Striatal GABA-MRS predicts response inhibition performance and its cortical electrophysiological correlates. Brain Struct. Funct..

[45] Kolasinski J (2019). The dynamics of cortical GABA in human motor learning. J. Physiol..

[46] Haag L (2015). Interrelation of resting state functional connectivity, striatal GABA levels, and cognitive control processes. Hum. Brain Mapp..

[47] Puts NAJ (2015). Reduced GABAergic inhibition and abnormal sensory symptoms in children with Tourette syndrome. J. Neurophysiol..

[48] Silveria MM (2013). Frontal lobe GABA levels during adolescence: associations with impulsivity and response inhibition. Biol. Psychiatry.

[49] Harris AD (2014). Impact of frequency drift on gamma-aminobutyric acid-edited MR spectroscopy. Magn. Reson. Med..

[50] Bell T (2020). In vivo Glx and Glu measurements from GABA-edited MRS at 3 T. NMR Biomed..

